# Agro-Morphological, Yield and Quality Traits and Interrelationship with Yield Stability in Quinoa (*Chenopodium quinoa* Willd.) Genotypes under Saline Marginal Environment

**DOI:** 10.3390/plants9121763

**Published:** 2020-12-13

**Authors:** M. Iftikhar Hussain, Adele Muscolo, Mukhtar Ahmed, Muhammad Ahsan Asghar, Abdullah J. Al-Dakheel

**Affiliations:** 1 International Center for Biosaline Agriculture (ICBA), Crop Diversification and Genetic Improvement Section, Dubai P.O. Box 14660, UAE; 2 Department of Plant Biology & Soil Science, Faculty of Biology, Campus As Lagoas Marcosende, University of Vigo, 36310 Vigo, Spain; 3 CITACA, Agri-Food Research and Transfer Cluster, Campus da Auga, University of Vigo, 32004 Ourense, Spain; 4 Department of Agriculture, Mediterranea University, Feo di Vito, 89122 Reggio Calabria, Italy; amuscolo@unirc.it; 5 Department of Agricultural Research for Northern Sweden, Swedish University of Agricultural Sciences, SE-901 83, Umea, Sweden; mukhtar.ahmed@slu.se; 6 Department of Agronomy, PMAS Arid Agriculture University, Rawalpindi 46000, Pakistan; 7 CAS Key Laboratory of Mountain Ecological Restoration and Bioresource Utilization & Ecological Restoration Biodiversity Conservation Key Laboratory of Sichuan Province, Chengdu Institute of Biology, Chinese Academy of Sciences, University of Chinese Academy of Sciences, Chengdu 610000, China; ahsanasghar2017@mails.ucas.ac.cn; 8 Research Farms, United Arab Emirates University, Al-Ain P.O. Box 15551, UAE; a.dakheel@uaeu.ac.ae

**Keywords:** salinity, *Chenopodium quinoa*, biomass, functional plant traits, biochemical traits, genotypes, yield, salt stress

## Abstract

Quinoa (*Chenopodium quinoa* Willd.) is a halophytic crop that shows resistance to multiple abiotic stresses, including salinity. In this study we investigated the salinity tolerance mechanisms of six contrasting quinoa cultivars belonging to the coastal region of Chile using agro-physiological parameters (plant height (PH), number of branches/plant (BN), number of panicles/plant (PN), panicle length (PL), biochemical traits (leaf C%, leaf N%, grain protein contents); harvest index and yield (seed yield and plant dry biomass (PDM) under three salinity levels (0, 10, and 20 d Sm^−1^ NaCl). The yield stability was evaluated through comparision of seed yield characteristics [(static environmental variance (*S*^2^) and dynamic Wricke’s ecovalence (*W*^2^)]. Results showed that significant variations existed in agro-morphological and yield attributes. With increasing salinity levels, yield contributing parameters (number of panicles and panicle length) decreased. Salt stress reduced the leaf carbon and nitrogen contents. Genotypes Q21, and AMES13761 showed higher seed yield (2.30 t ha^−1^), more productivity and stability at various salinities as compared to the other genotypes. Salinity reduced seed yield to 44.48% and 60% at lower (10 dS m^−1^) and higher salinity (20 dS m^−1^), respectively. Grain protein content was highest in NSL106398 and lowest in Q29 when treated with saline water. Seed yield was positively correlated with PH, TB, HI, and C%. Significant and negative correlations were observed between N%, protein contents and seed yield. PH showed significant positive correlation with APL, HI, C% and C:N ratio. HI displayed positive correlations with C%, N% and protein content., All measured plant traits, except for C:N ratio, responded to salt in a genotype-specific way. Our results indicate that the genotypes (Q21 and AMES13761) proved their suitability under sandy desert soils of Dubai, UAE as they exhibited higher seed yield while NSL106398 showed an higher seed protein content. The present research highlights the need to preserve quinoa biodiversity for a better seedling establishment, survival and stable yield in the sandy desertic UAE environment.

## 1. Introduction

Soil degradation due to salinity is a big issue in agriculture and forestry, especially in marginal environments. Several factors, such as scarce water resources, loss of topsoil due to wind erosion, sandy soils and high temperature in desert ecosystems are major constraints for crop production [1,2,3] affecting 250 million people [4]. Many arid and semi-arid regions of the world exhibit a significant portion of salt and degraded marginal lands. According to Wang et al. [4], approximately 20% of the land area is degraded. Global Assessment of Soil degradation (GLASOD) reported that 12 million heactares of land are degraded each year in arid ecosystems, at a cost to the global economy of up to US $42 billion per annum [5]. Furthermore, the continuous increase in global demand for food, fuel and feed has shifted the focus towards degraded lands, because land suitable for food production is shrinking worldwide [6]. This situation is further aggreviated by strong winds in arid and semi arid areas that cause erosion and land degradation, removing the top productive soil layers that can support plant growth [7].

Salinity is a complex phenomenon that critically impacts the morphological and physiological traits of crops through modifications in the osmotic balance, ion homeostasis, and reactive oxygen species regulation, each having a complex and less understandable genetic basis. High accumulation of salts in saline soils results into reduced water potential of soil solutions which causes difficulty for plants to extract water from soil experiencing “osmotic stress” [8]. Specific ion toxicity, the result of excessive uptake of certain ions (Na^+^ and Cl^−^) is the primary cause of growth reduction [2,9,10,11]. Toxic ions in salt-affected soils are usually sodium, chloride and sulphate [2,12]. The excessive Na^+^ accumulation causes ion toxicity and interferes with plant metabolism, while accumulation of K^+^ can alleviate Na^+^ toxicity by adjusting osmotic potential and through ion balance. Crop performance may also be adversely affected by salinity-induced nutritional imbalances [13,14]. These imbalances may result from the effect of salinity on nutrient availability, competitive uptake, transport and/or partitioning within the plant caused by physiological inactivation of a given nutrient resulting in increased plant’s internal requirement for that essential element [2,15,16]. One or more of these processes may occur at the same time, but whether they ultimately affect crop yield or quality depends on the level of salt stress, composition of salts, crop species, the nutrient in question and a number of environmental factors [15,16]. Therefore, it is imperative to advance our knowledge regarding the identification and evaluation of genotypes and landraces that can be cultivated in nutrient poor and sandy marginal soils. Moreover, these germplasms might have the potential for the restoration of these regions. Several studies concerning salinity tolerance in cultivated crops have been reported, while considerable advances have been made in the development of crop genotypes resistant to drought or salinity [17,18]. Therefore, salt-affected soils can be utilized by growing salt tolerant plants, whether halophytes or non-halophyte crops. The use of halophytic crop species can be considered as a valuable option to sustain agricultural production under saline and dry conditions and potentially under irrigation with saline waters [19,20]. However, it is imperative to explore intra-specific (inter-cultivar) variation for salt tolerance of a crop by screening its available germplasm.

Halophytes have shown potential to be useful resources for global food production, and contribute to the rehabilitation of salt-degraded lands. Quinoa (*Chenopodium quinoa* Willd.) is an important facultative halophyte, and its demand has increased recently in all continents [21]. Quinoa is a highly nutritious pseudo-cereal and has a wide potential to enhance food security through tolerating saline and marginal lands and to alleviate pressure on fertile agricultural soils [22]. Quinoa seeds are enriched in proteins, amino acids (lysine, methionine, threonine) [23]. Different fatty acids (linoleic and linole) and oleic acids have been reported in the seed oil [23]. From a human health perspective these fatty acids are of high quality as compared to those reported from other cereals [24]. Moreover, quinoa seeds are free from any allergic effects that may be caused by harmful chemicals or gluten, which is present in other cereals [25]. Quinoa exhibit various minerals like calcium, phosphorus, potassium, magnesium, phosphorus and zinc in sufficient quantity and protein contents (12–17%) [26]. The quinoa crop has been internationally recognized as a contributor to global food security because it is tolerant to many environmental constraints, such as frost [27,28], drought [29], and salinity [30]. In many developing African countries, quinoa has been significantly introduced in agro-ecosystem and has contributed to the regional food security [31,32]. However, evaluation of salinity tolerance potential of different quinoa genotypes and the possible evaluation of their growth, development and yield behaviour in salt-degraded marginal Arabian Peninsula Sandy desert soils are rarely studied.

The phenotypic plasticity, genotype variability and agronomic adaptation of quinoa are extremely wide and varied significantly from hot arid to subtropical humid climates [33]. Under these circumstances, it is viable to select, introduce, adapt and breed different quinoa genotypes in a wide range of environments. However, to increase food security and agriculture productivity in resource poor and degraded marginal lands like those of United Arab Emirates, it is imperative to study the salt tolerance potential of different quinoa genotypes and assess their yield stability without losing the grain quality. Jacobsen et al. [33], the quinoa plant photoperiod is a critical functional trait and should be evaluated before introducing it in a particular environment. Most of the previously referred studies have been carried out in quinoa germination and seedling growth responses under the control growth chambers. It is important to understand that germination and early seedling establishment stage is critical in the life cycle of plants, and many colleagues have documented these phenomena [34,35]. However, yield response factors of different quinoa genotypes to saline and marginal soil environment and their salinity tolerance mechanisms that may regulate seedling growth, yield and grain quality attributes need to be evaluated under marginal and nutrient poor sandy soils.

Studying the salinity tolerance potential of different quinoa genotypes and the elucidation of seed yield and yield stability is of paramount importance. Therefore, the evaluation of salinity tolerance potential of different quinoa genotypes was conducted under the hyper arid climate of Dubai, UAE with the following main objectives: (i) evaluation of the adaptation of accessions of different origins to the UAE sandy desert marginal environment with spring sowing, (ii) assessment of the salinity tolerance potential of six quinoa genotypes, (iii) determination of the variation and heritability of quinoa morphological and quality traits and their interrelationship with yield attributes and (iv) identification of the impact of saline water in growth, yield stability and grain protein content. The study will help to discriminate between resistant and sensitive quinoa genotypes and to understand the mechanisms of adaptation, for selecting genotypes tolerant to saline water irrigation in order to adapt to salt-degraded marginal sandy desert in the hyper arid UAE environment.

## 2. Material and Methods

### 2.1. Experimental Site

The study was carried out at the International Center for Biosaline Agriculture (ICBA) research station in Dubai (United Arab Emirates). The site has latitude of 25°13 N, a longitude of 55°17 E and is 16 m above mean sea level. The local climate is dominated by hot dry weather (April–October), with no rainfall at all during this transitional period and higher air temperature (exceeding 50 °C) during peak season (June, July, August) and high humidity. The winter season (December–February) is cool and dry. The field plot soil is a well drained sandy coarse (97% Sand, 2.2% Silt, 0.2% clay). The soil, classified as Carbonatic, Hyperthermic typic Torripsamment has an EC of 0.2 dS m^−1^. The soil physical and chemical properties are presented in Table 1 as the mean of two consecutive years (2013 and 2014).

### 2.2. Field Soil Physiological and Chemical Analysis

For the analysis of soil salinity, soil samples were taken from each of the sub-plot from 0–60 cm depth prior to the experiment and after harvesting the crop. The air-dried samples were analyzed for physiochemical analysis at the Central Analytical Laboratory, International Center for Biosaline Agriculture, Dubai (UAE). Standard soil analysis methods [36] were used and the results were presented on oven dried soil weight basis (Table 1). The electrical conductivity (E_C_) was measured in the soil extract collected from the saturated soil paste (dS m^−1^). Soil pH of the saturated paste was measured with a standard pH meter calibrated using buffer solutions (pH 4, 7 and 10). Soil texture was determined by using standard Pipette method and wet sieving and sand, silt and clay contents were used on USDA triangle to determine soil textural class. The total amount of nitrogen was determined using standard Kjeldahl method and phosphorous through colorimetri procedure. Available K was detected in 1 N ammonium acetate extract using a flame photometer. Organic carbon (OC) was measured through dichromate oxidation and converted to organic matter by multiplying by a factor of 1.72 [37].

### 2.3. Land Preparation, Sowing, Plant Growth and Agronomic Practices

**Figure 1:**
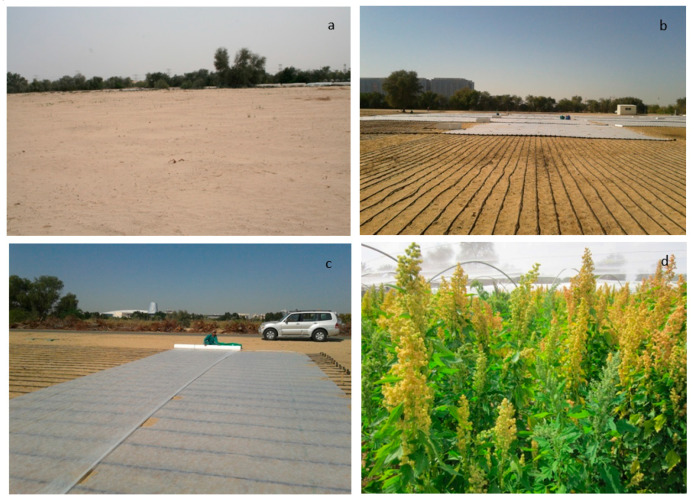
Photos to illustrate the sandy desert marginal soil of ICBA (**a**); irrigation system installation (**b**); quinoa seed sowing and covering with acryl sheet to protect from bird attacks (**c**); quinoa crop at panicle stage (**d**).

The experiemental soil was prepared by a disc plough, harrowed in order to obtain a good seed bed. The organic fertilizer (40 t ha^−1^) was applied and incorporated into the soil to improve the soil fertility. The chemical fertilizer {NPK (20:20:20)} at the rate of 50 Kg ha^−1^ was applied in two split doses by banding alongside the rows. Experimental design was a randomized complete block with split plot arrangement replicated three times. Quinoa genotypes (Table 2) were manually planted on 26 November 2013. Three salinity treatments (0, 10 and 20 dS m^−1^) were assigned to each main plot (the salinity treatments were applied one month after the seedling establishment), and six genotypes were assigned to the sub plots. Seeding was carried out by burying 3–4 seeds into the soil to a depth of 1–2 cm close to the dripper. Each genotype was planted in five rows (3 m long) per plot, 25-cm interplant spacing and 50 cm between the rows and one meter between two accessions. The plots were drip irrigated through out the study period with water of different salinities through drop laterals. The field was covered with acryl sheet, following sowing, to protect the plants from bird attacks (Figure 1). The seedlings were thinned to one plant per point, later, following plant establishment.

### 2.4. Irrigation System and Treatment Application

**Figure 2:**
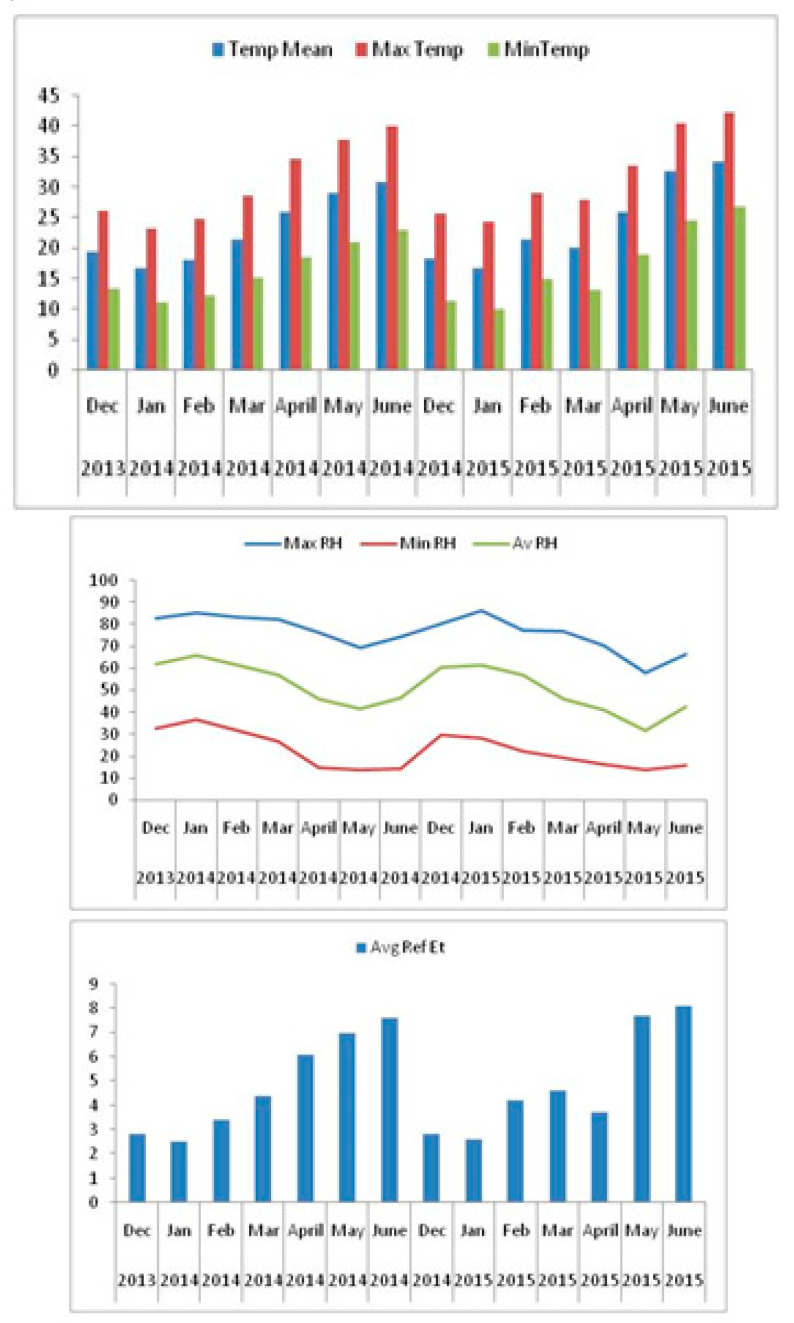
Monthly values of mean (T mean), maximum (T max), and minimum (T min) air temperature, relative humidity and reference evapotranspiration (ETo) in the ICBA weather station, Dubai, UAE.

During each growing season, saline water treatments (0, 10 and 20 dS m^−1^) were established and applied after one month from sowing (to ensure plant establishment) using drip irrigation system. Irrigation was supplied via a drip system (drip laterals of 16 mm in diameter), 0.25 cm emitters and were delivering 4.0 L h^−1^. The experimental plots were equipped with three irrigation valves from RainBird Company (Azusa, CA, USA). One valve was handling fresh, other saline and third valve (a solenoid valve of 2” size) controlling both saline and fresh water after the main control valve. The 3rd valve was controlling the irrigation water according to the main controller instructions. Saline irrigation water was provided from seedlings (one month old) to the grain filling stage. The climate data in terms of temperature, rainfall, wind speed and humidity was collected from weather station established at the ICBA (Figure 2).

### 2.5. Crop Phenology and Morphological Measurements

During the whole crop season, hand weeding was carried out when needed, without applying any herbicide. The data was collected from the middle 1 m of the two central rows. Following the completion of the physiological attributes, other morphological measurements were collected from five plants from each subplot. The average plant height (cm) from the ground level to the tip of the panicle on the main stem was measured at physiological maturity. The total number of branches from the main stem at different node positions, including the basal branches were recorded.

### 2.6. Harvesting, Biomass and Yield Traits

The yield data was collected at physiological maturity (when the seeds from panicle became hard [38]. From each plot, the number of panicles per plant was counted from five plants. The mean length (cm) of three panicles was taken randomly and averaged. The plant fresh biomass was recorded and then plant material was oven dried at 80 °C for three days and weighted. From each sub-plot, a sample line of 1 m length from the central rows was harvested and seeds were removed from the panicles of the plants, threshed, and weighed (g m^−2^) and then converted into t ha^−1^.

### 2.7. Leaf Carbon (%) and Nitrogen Contents (%) Analysis

The leaf samples from each treatment/plot and control were collected, oven dried and ground into a fine powder. Total N and C contents (% dry matter) were measured by elemental analysis, Flash EA-1112 (Thermo Fisher Scientific, Schwerte, Germany).

### 2.8. Harvest Index (%)

Harvest index was calculated by using the following formula:

Harvest index (%) = Grain yield/dry biomass × 100(1)

### 2.9. Grain Protein Contents Measurements

The grains (FW = 200 mg) from each genotype (three replicates/treatment) were crushed into liquid nitrogen and protein contents were measured using Commercial bovine seroalbumin (BSA) through Bradford assays [39] as reported by Hussain and Reigosa [40].

### 2.10. Statistical Analysis

The agro-physiological parameters were divided into two categories; yield and biochemical traits (carbon (C%), nitrogen (N%), seed yield (SY), harvest index (HI), grain protein contents); agro-morphological traits (plant dry matter (PDM), number of branches (BN), number of panicles (PN)). For each trait, the genotype-treatment combinations (i.e., six genotypes crossed with three salinity treatments) were subjected to analysis in order to summarize the relative merit of genotypic effects and growing conditions as causes of changes in the plant ecophysiological attributes and Dunnett test was employed as post-hoc test for multiple comparison. All analysis were conducted through General Linear Modeling (GLM) procedure and analysis of variance (ANOVA) using the SPSS for Windows version 17.0 software (SPSS Inc., Chicago, IL, USA). Difference between treatments means were compared using Tukey’s HSD test. A Pearson’s correlation matrix was conducted to assess the relative contribution of ecophysiological trait associations towards the seed yield at overall salinity.

A static yield stability index was calculated according to environmental variance (*S*^2^) [41]. Meanwhile, a dynamic yield stability index was presented following Wricke’s ecovalence (*W*^2^) [42].

## 3. Results

### 3.1. The Effect of Treatments and Genotypes on Growth Parameters

Quinoa plants were significantly shorter (69.44 cm) after treatment with salinity level of 20 dS m^−1^ as compared to the control (102.55 cm) (Table 3). Genotypes also showed a significant variation in PH that was higher in quinoa genotypes AMES 13,761 (120.44 cm), followed by Q21 (87 cm), Q29 (79.88 cm) and NSL106398 (77 cm) (Table 4). The Q22 showed PH of 73.33 cm (Table 4). The Q18 had the maximum number of branches/plant (19.0), followed by Q29 (15.66), Q21 (15.33) and AMES13761 (15.0) (Table 4). The lowest number of branches/plant was recorded in Q22 (14.11). Water salinity decreased the number of branches/plant that was significantly reduced at each salinity level as compared to control. The highest numbers of panicles/plant were observed in Q18 (17.44), followed by Q29 (14.22) and NLS106398 (13.56). Q21 and Q22 demonstrated in average a similar number of panicles (12), while the lowest number of panicles was produced by AMES13761 (10.56). Quinoa genotypes, AMES 13761, Q21, and Q29 produced longest panicles i.e., 20.78, 19.89, 18.78 cm respectively. Genotype Q18, Q22 and NSL106398 produced the smaller size panicle (17.0–17.74 cm). The application of S3 (20 dS m^−1^) caused retardation in number of panicles/plant. However, average panicle length was unaffected by salinity treatment. Quinoa genotype Q21 and Q22 produced the highest plant dry biomass (9.0 t/ha) followed by Q18 and Q29 that on average produced 6.28 and 6.74 t/ha. AMES 13,761 (7.94 t/ha) and NSL106398 produced on average 5.91 and 5.45 t/ha dry biomass, respectively (Table 4). Furthermore, plant dry biomass was unaffected by saline water treatment at all salinity level compared to control.

### 3.2. Saline Water Irrigation Impact on Carbon (C%) and Nitrogen (N%) and C/N Ratios

Genotypes differed for all traits, whereas a significant interaction existed between genotype and treatments (GT) for leaf N%, grain yield and harvest index (Table 3). The highest salinity treatment was lethal and affected all growth studied traits. There was significant difference in the leaf N% among the genotypes and NSL106398 exhibited the highest N% (2.23%), followed by Q18 (1.58%). Genotype Q29 showed the lowest N% (1.4%) in the dry leaf samples. The C% value was the highest in genotype Q21 (27.82%), followed by AMES13761 (27.16%) and Q18 (27.05%) (Table 4). The genotype Q22 exhibited the lowest values of C% (26.45%). Both salinity levels significantly decreased the C% as compared to the control. There was significant variation in C:N ratio values that were highest in Q22, Q29, AMES13761 and lowest in Q21. The results showed that N concentration was unaffected at all salinity treatments compared to control (Table 4).

### 3.3. Seed Yield and Harvest Index

The yield components (seed yield, harvest index) were all affected by salinity (Table 3). Genotypes Q21 and AMES13761 exhibited higher seed yield (2.30 t ha^−1^ and 1.77 t ha^−1^) than the other genotypes (Table 4). The lowest yield was produced by Q18 (1.27 t ha^−1^) that was 44.7% less than that of Q21 (Table 4). Salinity significantly decrease the seed yield that was 44.48% and 60% lower following exposure to 10 dS m^−1^ and 20 dS m^−1^, respectively (Table 3). Harvest index (HI) was significantly decreased following saline water treatment. Our results indicated that there was 34.53% and 60.38% reduction after 10 dS m^−1^ and 20 dS m^−1^ salinity treatment as compared to control, respectively (Table 3). Harvest index greatly varied among the genotypes and ranged between 31.8–19.05%. The highest HI values were observed in genotype AMES13761 followed by NSL106398 and the lowest in Q22. The genotype × treatment interaction (G × T) was also significant for seed yield and HI (Table 4).

### 3.4. Grain Protein Contents

There was significant impact of salt stress on grain protein contents and it was genotype dependent. The GP contents were the highest in NSL106398 (13.9 mg/g DW), followed by Q18 (9.89 mg/g DW), AMES13761 (9.19 mg/g DW) and Q22 (9.12 mg/g DW), respectively. The lowest GP was recorded in Q29 (8.76 mg/g DW) following salt stress treatment. There was a tendency of stimulation in GP following salinity treatment as compared to control (Table 3).

### 3.5. Yield Stability Trend in Quinoa Genotypes

The grain yield stability among different quinoa genotypes is shown in Table 5. The static environmental variance (*S*^2^) and dynamic Wricke’s ecovalence (*W*^2^) for different quinoa genotypes was different. *S*^2^ was in the range of 1.019–3.461.

However, a different trend was observed among six quinoa genotypes for *W*^2^ that varied from 1.67–9.58. In stability analysis, the lowest values demonstrate the stability in yield over saline environments. The variety ‘Q18’ was static stable and high yielder, ranking 1st for *S*^2^i grain yield index across all saline environments. The quinoa ‘Q22’, ‘Q29’ and ‘NSL106398’ were found to produce the the 2nd, 3rd and 4th highest static yield index among these tested genotypes and across all salinity treatments. The genotype ‘Q18’ showed stable mean yield (*W*^2^i) and ranked 1st among all the genotypes across all environments. Moreover, genotype ‘Q22’ was *S*^2^i and high yielder, ranking the 2nd for *W^2^i* grain yield index (Table 5).

### 3.6. Correlations between Seed Yield, Agro-physiological and Yield Attributes

Pearson’s correlations analysis showed significantly positive relationships between PH, TB, HI, C% with SY. However, significant and negative correlations were observed between N% and protein contents and seed yield (Table 6). The PH showed significant +ve correlation with AIL, HI, C% and C:N ratio. The NOB and NOI exhibited significant +ve correlation with NOI and AIL. The NOI showed + ve relation with AIL, N%, and protein contents. Harvest index displayed positive correlations with the C%, N% and protein content. N% exhibited +ve relation with C%, C: N ratio and protein contents.

## 4. Discussion

The rising global demand for nutritious and healthy food has challenged scientists to look for alternate crops, especially for the marginal areas where agricultural production is inefficient due to unfavorable climatic conditions, low soil fertility and lack of good quality irrigation water. In the Arabian Peninsula, scientists are experimenting with quinoa production because it is rich in nutrients, tolerant to salinity and uses much less water than other crops. Against this backdrop, this study identified the agro-morphological traits (PDM, BN, PN) that showed a decreasing trend with increasing salinity. Gómez-Pando et al. [13] showed that germination and plant height were highly decreased in quinoa plants that were subject to different salinity levels. Additionally, some genotypes (total 15 genotypes) were tolerant and less affected while others were susceptible and their agro-physiological attributes were decreased significantly following salinity treatment.

Under the saline and marginal UAE environment, some agro-physiological traits (PH, NBP, and NPP) were decreased after salt stress while PL was unaffected at various salinity levels. A decrease in dry matter yield with increasing soil salinity levels might be due to the inhibition of water availability and hydrolysis of reserved foods and their translocation to the growing shoots [43,44]. Other factors responsible for lower dry biomass yield may include panicle length, chlorophyll concentrations, number of productive tillers, number of primary branches per panicle, and fertility percentage [45]. The reduction in number and size of the panicles per plant is directly related to lower seed yield [46]. Plant biomass, height and seed yield, number of branches, number of panicles, panicle weight and harvest index were reduced in response to saline water irrigation and were subject to genotypic variation [8]. Genotypic variability in seed yield and biomass has been reported before for quinoa plants growing under comparable agroecological conditions [8,43,46]. Moreover, the significant interaction between genotype and irrigation conditions for seed yield, biomass and different agronomical traits highlights not only the genotypic plasticity available in the species but also the need to assess genotypic performance within each growing condition [47]. In the present study, PH was decreased after salinity treatment and AMES 13,761 was the tallest variety while Q22 was dwarf genotype. This was due to the stunted growth of plants caused by the high salt concentration in the nutrient medium. Adolf et al. [48] evaluated 14 quinoa genotypes at different salinity levels and recorded growth and biomass attributes. Pandela rosada and Utusaya were least affected and had capacity to be adapted to the harsh climate of southern altiplano of Bolivia (3600 m above sea level). Another quinoa variety, Amarilla de Maranganí was more tolerant and was not affected with respect to height and biomass production. The results of this study also suggest that plant morphological and agro-physiological characteristics are interrelated factors that highly impact the plant establishment, seedling growth, and yield. Meanwhile, the responses of different quinoa genotypes against salinity were different indicating their genetic diversity. According to different researchers, several mechanisms might contribute towards genotypic differences in salinity tolerance in quinoa. These mechanisms may include Na^+^ exclusion from leaf mesophyll cells, better H^+^ pumping to restore membrane potential, and Na^+^ exclusion from leaf cells demonstrating salinity tolerance of quinoa [32,33,49]. The information about these functional attributes might facilitate the restoration programs of degraded marginal salt affected soils and will help to convert waste to wealth assets.

The evaluation and selection of salt tolerant quinoa genotypes is an important step to pursue their adaptation uder marginal and sandy soils and to check the effect of salinity on grain yield and grain protein contents. The present results demonstrated that significant genetic diversity exists between different quinoa genotypes. Yield components like NPP and PL were different among genotypes. Salinity significantly affected the number of panicles per plant and average panicle length also varies from one to other genotype. According to the report of Long Nguyen [50]; panicle length in quinoa is interconnected with grain yield and variation in this trait lead to significant variation in the final yield. Several researchers have noticed that long panicle bearing genotypes demonstrated higher yield than genotypes with shorter panicles [45,46]. From the results of present study, it was concluded that differences in panicle length were connected with genotypic difference rather than salinity impact.

The seed yield was significantly decreased (60%) at high salinity. It is a typical phenomenon of plants affected by environmental stresses that showed a restricted supply of CO_2_ as well as reduced activity of RuBisCO. These processes leads to reduced photosynthesis, carbon assimilation, growth and yield of the plants [16,51,52,53,54]. Previously published data showed that the small panicle length, chlorophyll concentrations, number of productive tillers, and lower number of primary branches per panicle were responsible factors in the low yield of quinoa [45].

The salinity treatment did not affect very much the leaf C and N. However, the allocation of N and C was different among the different genotypes and changed in respect to the genetic variability. Grain protein contents (GP) differed significantly after salinity treatment that was stimulated to a cetain extent. Genotype, NSL106398 showed higher grain protein contents while the lowest was observed in Q29. The reduction in quinoa plant dry biomass was 23.7% and 36% after treatment at 10 and 20 dS m^−1^ respectively. The growth of quinoa (cv. Hualhuas) was slightly increased following salinity treatment [55]. The present results demonstrated the competitive advantage of salinity tolerant quinoa genotypes in terms of morphological and ecophysiological attributes [6,13,18,21,43,45,56].

Our results indicate a difference in grain and biological yield (harvest index) among quinoa genotypes, showing that Q21 had a higher seed yield, followed by AMES13761, and both genotypes showed a typical genetic variation. Adolf et al. [48] reported similar results. They found that quinoa genotype, Utusaya (Bolivian origin) had high stomatal conductance compared to control plants and showed a reduction (25%) in CO_2_ while “Titicaca” (from Denmark) demonstrated higher reduction (67%) in CO_2_ assimilation. To counteract the salinity, Utusaya variety possessed some genetically improved salt tolerance mechanism (osmoprotectants) and water loss through transpiration was much less than other genotypes. Recent studies highlighted that some adaptation mechanisms exist in certain quinoa genotypes that control transpiration and thus, WUE, under saline conditions. Lately, it was correlated with morphological features (stomatal size reduction, density or both) [22,23,47,48,49]. A significant variation was also oberved in HI among genotypes while AMES13761 showed higher HI, followed by NSL106398 and it was lowest in Q22. Morover, salinity also reduced HI after treatment at 10 dS m^−1^ and 20 dS m^−1^. These results demonstrated a greater adaptability of quinoa genotypes to the agro-climatic conditions of UAE. Other researchers also documented that Chilean varieties were less sensitive to photoperiod and hence more adaptable to saline and marginal environments [27,28,29].

The correlation between diggerent physiological, yield and quality characteristics of quinoa are presented in Table 6. Several parameters were positively correlated with other contributing attributes while some also have negative correlation. More prominent +ve correlation was found betweeen NOB, NOI and AIL, TB, C:N ratio). The correlation among grain yield, biological yield and protein contents of grains is often misleading. This can lead to wrong conclusions and policy guidlines for future breeding strategies for marginal environments [57]. Confusion was largely provoked by the fact that the relations between biomass, number of panicle and yield were positive or negative, and sometimes there was no correlation depending on the crop and growing conditions.

### Lessons from Different Stress Levels: Trade-off between Survival and Growth

In the present study, the Chilean-based quinoa genotypes displayed remarkable variation in plant establishment, seedling growth, biomass yield, and panicle attributes and salinity responses yield potential. Different salinity treatments (10, 20 dSm^−1^) caused a significant reduction in biological and grain yield. Even though seed yield was reduced, quinoa was still able to perform relatively well under these sandy, nutrient poor and marginal soil conditions as compared to other high productive soil environments. Under low salt stress (10 dSm^−1^), average panicle length of quinoa varieties did not differ in their responses to salinity. However, at high salinity (20 dSm^−1^), seed yield was not highly reduced in all the tested varieties. In this regard, Q21 was highest yielder while Q18 was the most affected with lowest seed yield. We speculate that Q18 employed a “survival” strategy with a more reduced growth rate, biological yield (HI) but higher number of branches and number of panicles and medium level of leaf C% and N%. The grain protein contents were comparative to AMES13761 and Q21 but its seed yield was highly reduced at overall salinity, compared to other varieties. These adaptations allowed Q18 to survive longer, but at the trade-off of the very high reduction in growth rate and seed production. Several authors documented that there is great variation in salinity tolerance among quinoa genotypes [22,23,43,47,48,49,58,59,60]. Previously, it was assumed that only Bolivian Salares originated genotypes are high salt tolerance while, now, it is well known that salinity tolerance does not related with geographic distribution and genotypes from Chile coastal areas and highlands are even more salt tolerant [58,59,60]. Furthermore, it was recently reported that a wild relative of quinoa (*Chenopodium hircinum*) was found to have a much higher salinity tolerance level than quinoa cultivars [59]. Photoperiod and temperature attributes also played a significant role in the growth, development and final grain yield of quinoa. Some researchers demonstrated that quinoa genotypes originated from dry and cold environments were sensitive to the temperature as compared to varieties originated from hot and humid climates [38]. It was evidenced previously that solar rays affects phyllochron in the quinoa varieties. Thus varieties from these regions (Peru, Southern Chile and Bolivia) were more sensitive to solar radiation than Ecudorian varieties. While, it was demonstrated that Ecuadorian quinoas were more sensitives to photoperiod becuase they posess longest phyllochron. However, in the present studies, we did not observe any growth and yield loss due to photoperiod and most of the yield reduction was due to nutrient poor sandy soils and salt stress.

Salinity tolerance evaluation of quinoa indicated a significant diversity in growth strategies, agro-physiological attributes, yield stability and salinity coping phenomena’s that will assist in the restoration of sat-degraded marginal sandy soils. Quinoa as a highly nutritious crop and can be a potential candidate in nutrient poor sandy and salt-degraded marginal soils in arid and semi arid regions and will help to adapt the harsh environment, as well as also offer food security. The present studies also suggest the supply of essential plant nutrients through chemical fertilizers and organic manures as soil amendments may be a possible strategy to increase nutrient availability and soil water holding capacity in salt-degraded marginal sandy soils. Other management strategies could include strong seeding establishment at the start of the sowing using fresh water encouraging seedling survival for later irrigation with saline water under nutrient poor, sandy and vulnerable environment.

## 5. Conclusions

This study concluded that agro-physiological and yield stability attributes can be used to choose quinoa genotypes of contrasting performance across different saline environment and provide a platform to select certain quinoa genotypes for a broad or a specific adaptation. Overall, seedling performance throughout the growth and yield period were genotype dependent. Results highlighted that Chilian based quinoa genotypes showed sufficient adaptation under nutrient poor marginal soils and harsh environment of UAE and were not sensitive to photoperiod conversely to other quinoa genotypes. These genotypes might be useful for the rehabilitation of Dubai marginal soils. Therefore, we suggest that regional farmers might use identified and optimally adapted genotypes of quinoa, crop management strategies and adequate agronomic practices that can help to recover and use these marginal lands for sustainable crop production and food security. This study can be useful for selection, breeding, and up scaling quinoa genotypes in the Arabian Peninsula.

## Figures and Tables

**Table 1:** Soil classification, chemical and physical properties from 0–60 cm depth os soils at pre-sowing 2013 and post-harvest 2014 from each sub-plot, irrigated with 10 and 20 dS m^−1^.

Parameters	Values	
Pre-Sowing 2013	Post-Harvest 2014	Post-Harvest 2014
-	10 dS m^−1^	20 dS m^−1^
Sand (%)	97.60	97.60
Silt (%)	2.20	2.20
Clay (%)	0.20	0.20
Soil textural class	Sand	Sand
Ece dS m^−1^	2.04	4.10
pHs	7.04	7.31
Total N mg kg^−1^	52.00	51.59
P mg kg^−1^	41.51	46.74
K mg kg^−1^	45.95	41.61
%Organic matter	1.46	1.32

Ece: Electrical Conductivity.

**Table 2:** A list of six quinoa (*Chenopodium quinoa* Willd.) genotypes native to the Chilian coastal region that were used to identify ecophysiological indicators suitable for quantifying seedling salinity responses.

S. No.	Code	Germination Line	Source	Origin	Status	Seed Color
**2**	**Q 18**	*C. quinoa PI614886*	USDA	Maule, Chile	Cultivated	Yellow
**4**	**Q 21**	*C. quinoa PI614889*	USDA	Bio-Bio, Chile	Cultivated	Orche
**5**	**Q 22**	*C. quinoa PI634918*	USDA	Chile	//	Yellow
**8**	**Q 29**	*C. quinoa PI634925*	USDA	Chile	//	Yellow
**11**	**AMES 13761**	*C. quinoa*	USDA	USA	-	-
**12**	**NSL 106398**	*C. quinoa*	USDA	USA	-	-

**Table 3:** Genotype and treatment effects on biomass and agro-physiological traits, and yield components of six quinoa genotypes grown under different water salinity levels.

Variables	Plant Height (cm)	Number of Branches/Plant	No. of Panicles/Plant	Average Panicles Length (cm)	Plant Dry Biomass (t/ha)	Leaf N%	Leaf C%	C:N Ratio	Grain Protein Content	SY (t/ha)	HI
S1–0 (Control)	102.55a	16.88a	14.56a	18.23a	6.86b	1.41a	28.46a	13.73a	8.87c	2.63a	37.56a
S2–10 dS m^−1^	84.22b	14.72b	12.39c	18.57a	6.64b	1.61a	26.54b	7.94b	10.06b	1.46b	24.59b
S3–20 dS m^−1^	69.44c	15.33b	13.5b	18.85a	6.71a	1.75a	25.97b	8.4b	10.98a	1.06c	14.88c
Genotype (G)	0.00	0.29	0.05	0.22	0.00	0.00	0.265	0.105	0.000	0.04	0.012
Treatment (T)	0.00	0.35	0.35	0.88	0.36	0.00	0.00	0.00	0.00116	0.00	0.00
G × T interaction	0.79	0.296	0.35	0.48	0.37	0.00	0.91	0.85	0	0.02	0.016

Genotype values are the mean of nine measurements (three treatments and three replications per treatment), while treatment values are the mean of the 54 measurements (six genotypes and three replications per genotype). Means followed by different letters are significantly different (*p* ≤ 0.05) according to Tukey’s honestly significant difference (HSD) test. Treatments: S1—0, (control); medium salinity—S2, 10 dS m^−1^; high salinity—S3, 20 dS m^−1^; ns, not significant; G, Genotypes; T, Treatment.

**Table 4:** Genotype and treatment effects on biomass and agro-physiological traits, and yield components of six quinoa genotypes grown under different water salinity levels.

Variables	Plant Height (cm)	Number of Branches/Plant	No. of Panicles/Plant	Average Panicles Length (cm)	Plant Dry Biomass (t/ha)	Leaf N%	Leaf C%	C:N Ratio	Grain Protein Content	SY (t/ha)	HI
Genotypes (G)	-	-	-	-	-	-	-	-	-	-	-
Q 18	74.88d	19.0a	17.44a	17.0b	6.74b	1.58b	27.05a	9.88c	9.89b	1.27b	20.19c
Q 21	87.0b	15.33b	12.88c	19.89a	9.0a	1.43c	27.82a	8.04cd	8.96c	2.3a	26.98b
Q 22	73.33d	14.11c	12.22c	17.12b	9.01a	1.45c	26.45b	11.97a	9.12b	1.53b	19.05c
Q 29	79.88c	15.66b	14.22b	18.78a	6.28b	1.4c	26.76b	10.33b	8.76c	1.72b	26.04b
AMES 13761	120.44a	15b	10.56d	20.78a	5.91c	1.47c	27.16a	10.56b	9.19b	1.77b	31.8a
NSL 106398	77.0c	14.78c	13.56b	17.74b	5.45c	2.23a	26.74b	9.35c	13.9a	1.69b	30a

Genotype values are the mean of nine measurements (three treatments and three replications per treatment), while treatment values are the mean of the 54 measurements (six genotypes and three replications per genotype). Means followed by different letters were significantly different (*p* ≤ 0.05) according to Tukey’s honestly significant difference (HSD) test. Treatments: S1—0, (control); medium salinity—S2, 10 dS m^−1^; high salinity—S3, 20 dS m^−1^; ns, not significant; G, Genotypes; T, Treatment.

**Table 5:** Environmental variance (*S*^2^i) and Wricke’s ecovalence (*W*^2^i) over the ambient treatments and three climate treatments for the six quinoa genotypes with highest averaged mean yield across treatments (mi).

S. No.	Genotypes Name	Mi	*S*^2^i	*W*^2^i
1	Q 18	1.274	1.019	1.677
2	Q 21	1.082	3.461	2.080
3	Q 22	1.919	2.310	2.470
4	Q 29	1.494	2.591	2.349
5	AMES 13761	1.398	2.659	5.362
6	NSL 106398	2.577	2.540	9.586

**Table 6:** Pearson’s correlations among physiological and seed yield traits of quinoa as a result of genotypic collective response for all three salinity levels.

Traits	SY	PH	NOB	NOP	APL	TB	HI	N%	C%	CN Ratio	Protein
SY	1	-	-	-	-	-	-	-	-	-	-
PH	0.46 **	1	-	-	-	-	-	-	-	-	-
NOB	−0.001	0.212	1	-	-	-	-	-	-	-	-
NOI	−0.039	0.034	0.832 **	1	-	-	-	-	-	-	-
AIL	−0.118	0.414 **	0.267 **	0.223 **	1	-	-	-	-	-	-
TB	0.209	−0.071	−0.026	0.08	0.058	1	-	-	-	-	-
HI	0.837 **	0.503 **	−0.012	−0.107	−0.167	−0.268 **	1	-	-	-	-
N%	−0.311 **	−0.201	0.136	0.244 **	0.043	−0.174	−0.249 **	1	-	-	-
C%	0.637 **	0.422 **	0.176	0.141	−0.031	0.025	0.637 **	−0.219 **	1	-	-
CN Ratio	0.119	0.264 **	0.051	0.019	0.01	−0.153	0.186	−0.277 **	0.182	1	-
Protein	−0.311 **	−0.201	0.136	0.244 **	0.043	−0.174	−0.249 **	0.000 **	−0.219 **	−0.277	1

** Correlation significant at *p* ≤ 0.05 according to Tukey’s HSD test; SY: Seed Yield, PH: Plant Height, NOB, Branch number; NOP: Panicles number, APL: Average Panicles Length, TB: Total Biomass, HI: Harvest Index, N%, Nitrogen concentration; C%, Carbon concentration; Protein: Grain Protein contents.
